# Dynamic response analysis of a whole steel frame solar greenhouse under wind loads

**DOI:** 10.1038/s41598-022-09248-z

**Published:** 2022-03-25

**Authors:** Xiaoye Li, Cong Wang, Yingchun Jiang, Yikui Bai

**Affiliations:** 1 College of Water Conservancy, Shenyang Agricultural University, Shenyang, China; 2 College of Horticulture, Qingdao Agricultural University, Qingdao, China; 3 College of Engineering, Shenyang Agricultural University, Shenyang, China

**Keywords:** Civil engineering, Fluid dynamics

## Abstract

In recent years, whole steel frame steel greenhouses have become increasingly prevalent. With the characteristics of large flexibility and small mass, whole steel frame steel greenhouses are sensitive to wind loads. However, studies on the safety of whole steel frame steel greenhouses under wind loads are still limited. In this study, a 10 m span whole steel frame solar greenhouse was taken as the research objective. Taking the Davenport spectrum as the target spectrum, the time history of the wind speed was simulated by the harmonic superposition method. The finite element model of the greenhouse structure was established using ANSYS software. The simulated wind pressure was applied on the greenhouse structure for dynamic response analysis. The dynamic response results were compared with the static analysis results under average wind load. The results showed that the greenhouse structure mainly bears bending stress under wind load. The bending stress, axial stress and displacement of the greenhouse skeleton under average wind loads are lower than those under instantaneous wind loads. It is necessary to consider the dynamic characteristics of wind loads in the design of solar greenhouses. A wind-induced vibration coefficient is obtained, which can be used to convert the dynamic load into the equivalent static load and improve its design efficiency.

## Introduction

Chinese solar greenhouses exhibit good thermal insulation and heat storage performance. These structures are widely used in agricultural production in northern China. Compared with traditional building structures, greenhouse structures are light and sensitive to wind and snow loads^1^. In recent years, a variety of extreme weather events have increased, such as rainstorms, snow, freezing temperatures, and strong winds^2^. Wind is a frequent and severe disaster in greenhouse production, that can damage greenhouses and cause substantial economic losses^3,4^. The structural safety of greenhouses is the precondition to ensure the normal production of greenhouses. Many researchers have studied the stability of greenhouse structures under various load cases^5–7^. Emekli et al.^8^ used SAP2000 software to analyze the functional characteristics of five types of greenhouses used in Turkey under static and dynamic loads. Using a large-sized wind tunnel, the wind pressure distribution and local damage of a large-span circular arch greenhouse built in coastal areas were studied^9^. It is suggested that the damage caused by the bending moment due to a strong wind load should be considered in the calculation of the wind pressure coefficient in structural design^10^. Briassoulis et al.^11^ established a three-dimensional structure model and a two-position frame model of a large multi-span circular arch greenhouse. Linear, geometric and material nonlinear analysis methods have been used to study the failure analysis of greenhouses under wind and snow loads. These studies provide references for the stability of greenhouse structures under various loads.

China has 3.7 million hectares of protected horticulture, accounting for 80% of the world. Solar greenhouses account for 30.5% of China's horticultural facilities^12^. When the greenhouse is designed in accordance with the code, the wind load is applied on the greenhouse structure as a static load. However, the greenhouse bears different amplitudes of dynamic wind loads. The dynamic response mechanism of greenhouse structures under dynamic loads needs further theoretical research. In our previous work, a block method was proposed for the dynamic response analysis of a solar greenhouse with a plane rigid frame model under wind load^13^. The wind-induced vibration responses of a 12 m span truss solar greenhouse were also investigated by the time domain method^14^. These two results showed that both the stress and displacement under instantaneous wind loads are larger than those under the corresponding average wind loads. Therefore, it is necessary to consider the amplification effects of fluctuating wind loads in the wind resistant design of solar greenhouses.

A traditional solar greenhouse is mainly composed of north walls (brick or earth walls) and steel truss structure^15^. The existence of walls gives the greenhouse better thermal insulation and heat storage capacity. However, the construction cost of traditional solar greenhouses is high, and the construction of walls also takes much time. With the development of the theory of active heat storage and release, a whole steel frame solar greenhouse has been proposed in recent years^16^. This greenhouse structure is composed of a south roof, a north roof and columns. In this type of greenhouse, walls are replaced by columns. Active heat storage and release equipment is used to compensate for the heat loss at night^17^. Instead of a truss structure, the whole steel frame structure solar greenhouse adopts a solid web steel structure, which has the advantages of simple modeling, convenient processing, better anticorrosion properties and fast installation^7,18,19^. The greenhouse is a single arch solid web structure, and the whole greenhouse is constructed of thin-walled galvanized steel pipe. To date, the related research studies have been mainly focused on the truss structure of traditional solar greenhouses. There has been little theoretical analysis on the whole framework greenhouse structures. Only Wang et al.^20^ studied the static properties and failure mechanisms of this type of greenhouse. However, this study only established a single plane model. Spatial analysis of the whole structure is lacking, and the effect of tie bars on the stability of the greenhouse structure is ignored.

Therefore, the aim of this study is to investigate the dynamic responses of a three-dimensional solar greenhouse structure considering fluctuating wind loads. In this research, the Davenport spectrum adopted by the China building structure load code is used as the target wind spectrum. The time scale curve of the fluctuating wind speed is simulated by harmonic superposition method. Taking the 10 m span of all steel frame solar greenhouses as the research objective, a three-dimensional finite element model of the greenhouse was established by using ANSYS software. The stress, stability and static force of the greenhouse under fluctuating and average wind loads were compared and analyzed. The safety of the structure was evaluated. It is hoped that this research can provide a reference for the analysis and design of whole frame solar greenhouses.

## Description of the whole frame solar greenhouse structure

**Figure 1:**
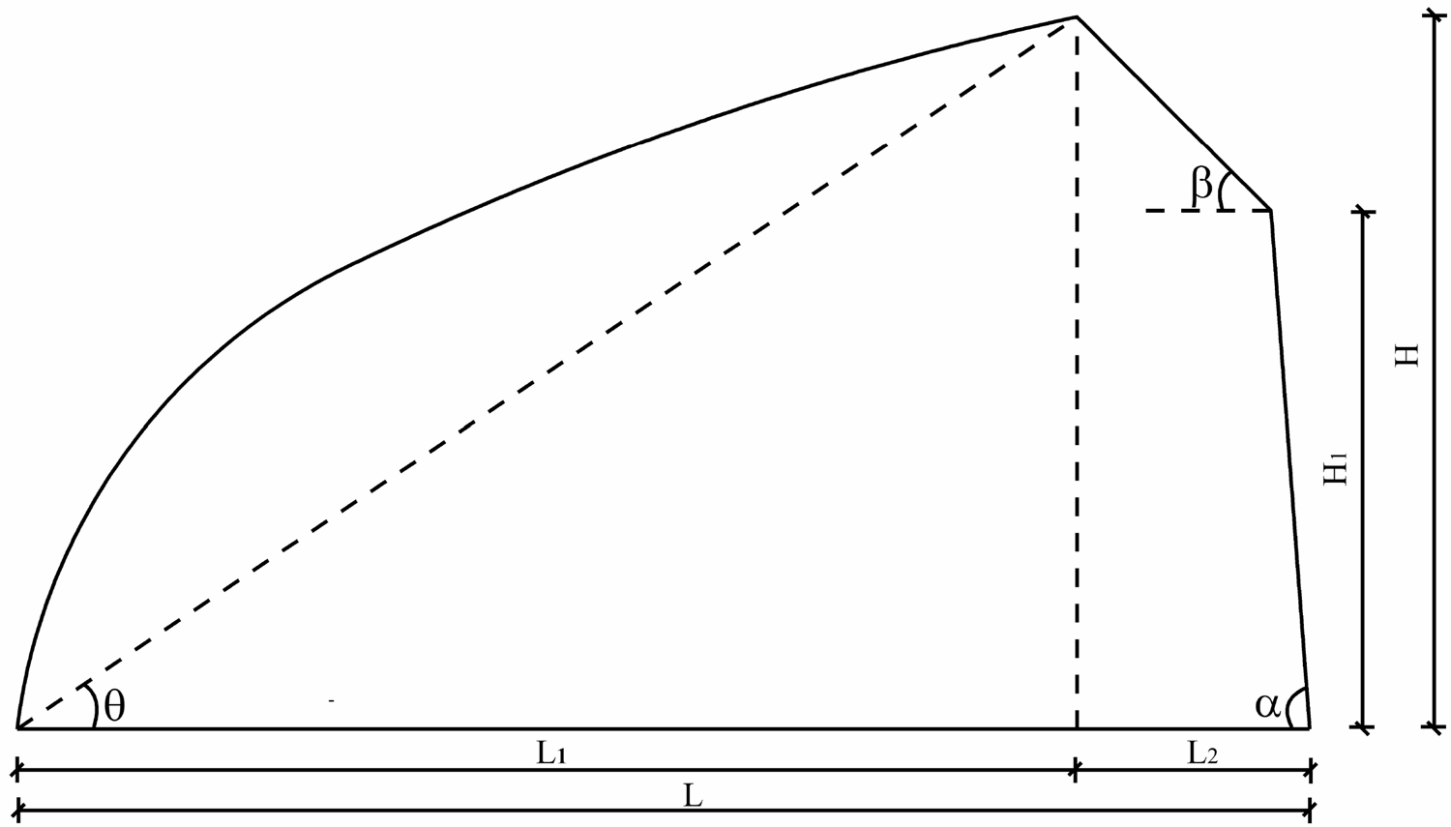
Section size of greenhouse.

The reference greenhouse is located in Tonghua (41.50°N, 125.50°E) Jilin, China. The section diagram of the greenhouse is shown in Fig. 1. And the greenhouse section parameters are shown in Table 1.

**Table 1:** Parameters of greenhouse section.

Span (*L*)/m	South roof angle (*θ*)/(°)	North roof angle (*β*)/(°)	Column angle (*α*)/(°)	South roof horizontal projection (*L*_1_)/m	North roof horizontal projection (*L*_2_)/m	Rear column height (*H*_1_)/m	Height (*H*)/m
10	34	45	86	8.2	1.5	4	5.5

**Figure 2:**
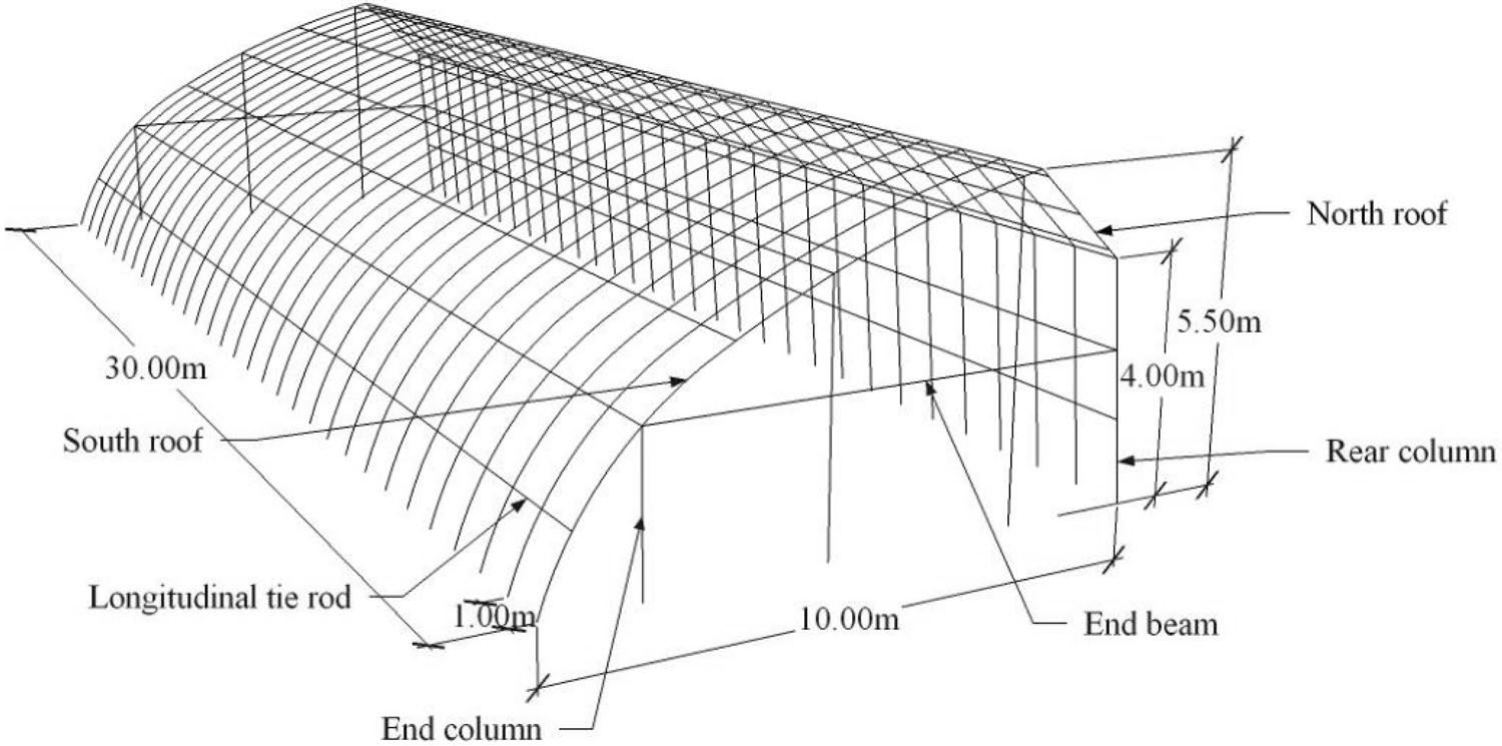
Schematic diagram of the greenhouse framework.

The space between each frame of greenhouse is 1 m, and the total length of the greenhouse is 30 m. The frames are connected by 13 longitudinal tie rods, 6 on the south roof, 3 on the north roof, 2 on the rear column, 1 on the connection between the north and south roof, and 1 on the connection between rear column and north roof. Longitudinal tie rods not only can transfer loads, but also provide out-of-plane stability. The schematic diagram of the greenhouse framework is shown in Fig. 2. The south roof frame and the north roof frame, the north roof frame and the rear column, and the main frame and the longitudinal rod of the greenhouse are fixed by fasteners. The bottom of the south roof skeleton and the bottom of each column are welded on the foundation embedded parts.

All the framework are made with Q235 thin-wall galvanized steel pipe, the material parameters are shown in Table 2, and the skeleton section of each part is shown in Table 3.

**Table 2:** Parameters of materials.

Material	Density/kg·m^−3^	Elasticity modulus /GPa	Poisson ratio	Yield strength /MPa
Steel Q235	7850	206	0.3	235

**Table 3:** Cross-sectional dimensions of materials.

Component	Section type	Section size /mm
South roof	Rectangular tube	90 × 35 × 3
North roof	Rectangular tube	90 × 35 × 3
Rear column	Rectangular tube	90 × 35 × 3
End column	Circular tube	30 × 2
End beam	Circular tube	30 × 2
Tie rod	Circular tube	30 × 2

## Finite element model

Based on the finite element analysis method, ANSYS software was used to simulate the whole framework solar greenhouse structure. For the solar greenhouse covered by plastic film, the load acting on the plastic film is redistributed to the structural frame^21^. Therefore, the model includes all steel frames, but does not include the plastic film. All the frame structures of the greenhouse are simulated by BEAM188 element. Based on Timoshenko beam theory, BEAM188 element is a three-dimensional linear beam with two nodes. Each node has 6 or 7 degrees of freedom. It is suitable for the analysis of slender beams and can calculate the bending moment and shearing force of the structure. Finite element models of greenhouses are established and meshes based on the structural parameters of each part.

## Wind pressure calculation

### Time history simulation of wind speed

The instantaneous wind consists of two components: one is the long-period part, which is more than 10 min; The other is the short period part, which is only a few seconds to tens of seconds^22^. Usually, the long period part is far away from the natural vibration period of greenhouse structures, and its usually equivalent to static loads. While the short period part is close to the natural vibration period of the structure, so its action has dynamic properties. According to the characteristics of wind load, the wind load acting on the structure is usually regarded as the combined action of average (static wind) and fluctuating wind.

The wind speed *V* (*x, y, z, t*) can be expressed by the sum of average wind speed $\overline{v} (z)$ and fluctuating wind speed *v* (*x, y, z, t*) as follows^23^:

1 $$ V(x,y,z,t) = \overline{v} (z) + v(x,y,z,t) $$

The average wind speed $\overline{v}{(z)}$ is affected by the change of ground height. The exponential rate distribution function can be used to describe the change of average wind speed in the near ground layer as follows^22^:

2 $$ \frac{{\overline{v} (z)}}{{\overline{v}_{{_{10} }} }} = (\frac{z}{10})^{\alpha } $$

where $\overline{v}_{10}$ is average wind speed at 10 m height, *z* is the height of the calculation point, *α* is the power-law exponent and is taken to be 0.16 in this study.

Fluctuating wind is caused by turbulence in wind flow, and its velocity variation is random. A large number of measured and analyzed results showed that it is a stationary random process with zero average value. Fluctuating wind speed is usually described by power spectrum and correlation function. The power spectrum of fluctuating wind speed mainly reflects the energy distribution of various frequency components in fluctuating wind. Wind speed spectrum can be divided into horizontal gust power spectrum, vertical gust power spectrum and transverse gust power spectrum. Vertical gust and transverse gust turbulence have little effects on wind speed, and the dynamic responses of structure are negligible. Therefore, only the horizontal gust power spectrum is considered in this study. Davenport established the empirical mathematical expression of fluctuating wind speed spectrum based on the strong wind records at different locations and heights in the world as follows^24^:

3 $$ S_{{\text{v}}} (n) = 4k\overline{v}_{10}^{2} \frac{{x^{2} }}{{n(1 + x^{2} )^{4/3} }} $$

where *S*_*v*_(*n*) is the power spectrum of fluctuating wind, m^2^/s; *k* is the surface roughness coefficient; $\overline{v}_{{10 }}$ is the average wind speed at the height of 10 m; *n* is the frequency of fluctuating wind, Hz; *x* is the integral scale coefficient of turbulence with the value of 1200*n*/$\overline{v}_{{10}}$.

Taking Davenport spectrum as target spectrum, the wind speed was simulated by harmonic superposition method. The harmonic superposition method is a spectrum representation method based on the sum of trigonometric series, which approximates the stochastic model of the target stochastic process by discrete spectrum.

**Figure 3:**
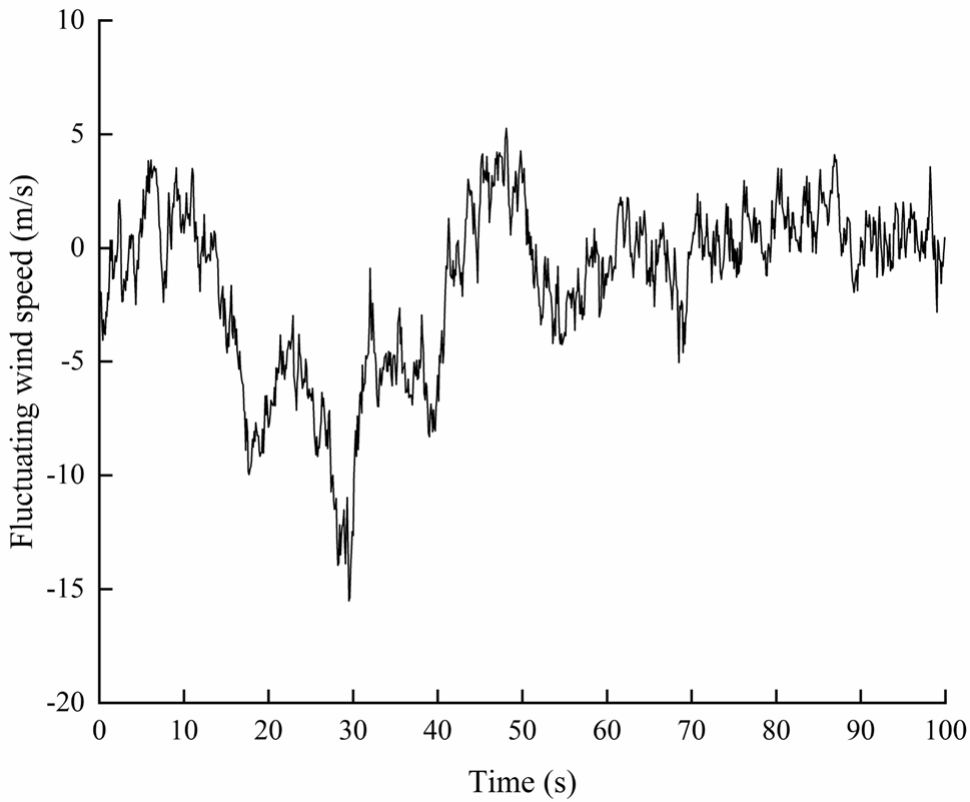
Fluctuating wind speed.

**Figure 4:**
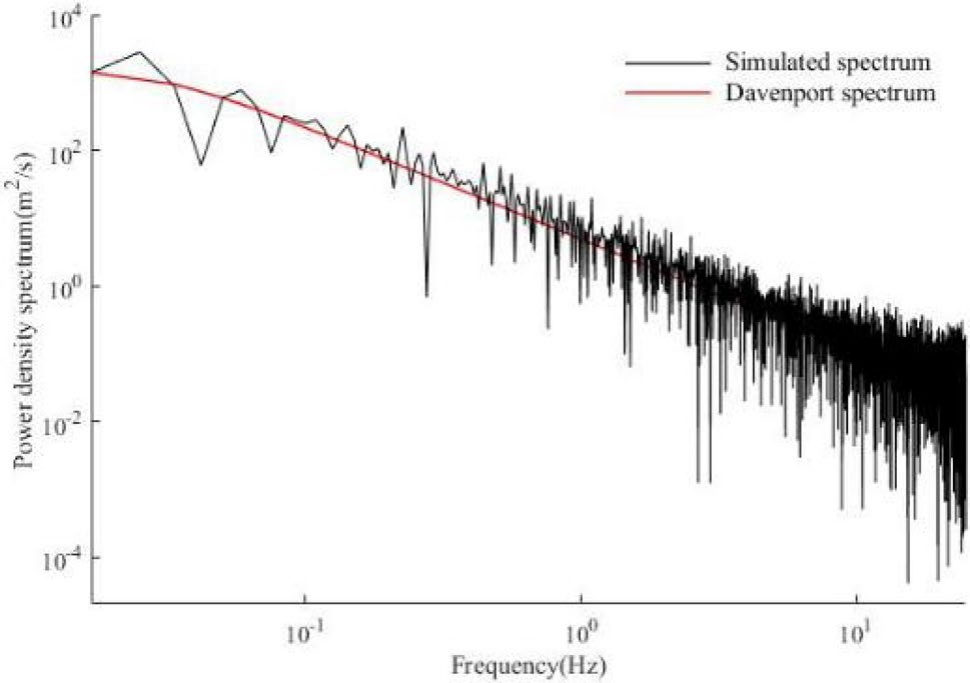
Comparison between simulated spectrum and Davenport spectrum.

Based on the above algorithm, the wind velocity of horizontal gust is simulated. The simulation time interval is 0.1 s, and the total time is 100 s. The average wind speed at 10 m height in Tonghua is 23.6 m/s. The time history of fluctuating wind speed shown in Fig. 3 is obtained, and the power spectral density curve of simulated wind speed is shown in Fig. 4. It can be seen that the power spectrum is in accordance with the target self-power spectrum, which shows that the simulation of the time history of fluctuating wind speed is reliable.

### Calculation of wind pressure on greenhouse structure

In order to investigate the influence of wind load on the safety of greenhouse structure, two load cases are considered in this research: (1) average wind load calculated based on average wind speed; (2) instantaneous wind load calculated based on instantaneous wind speed.

**Figure 5:**
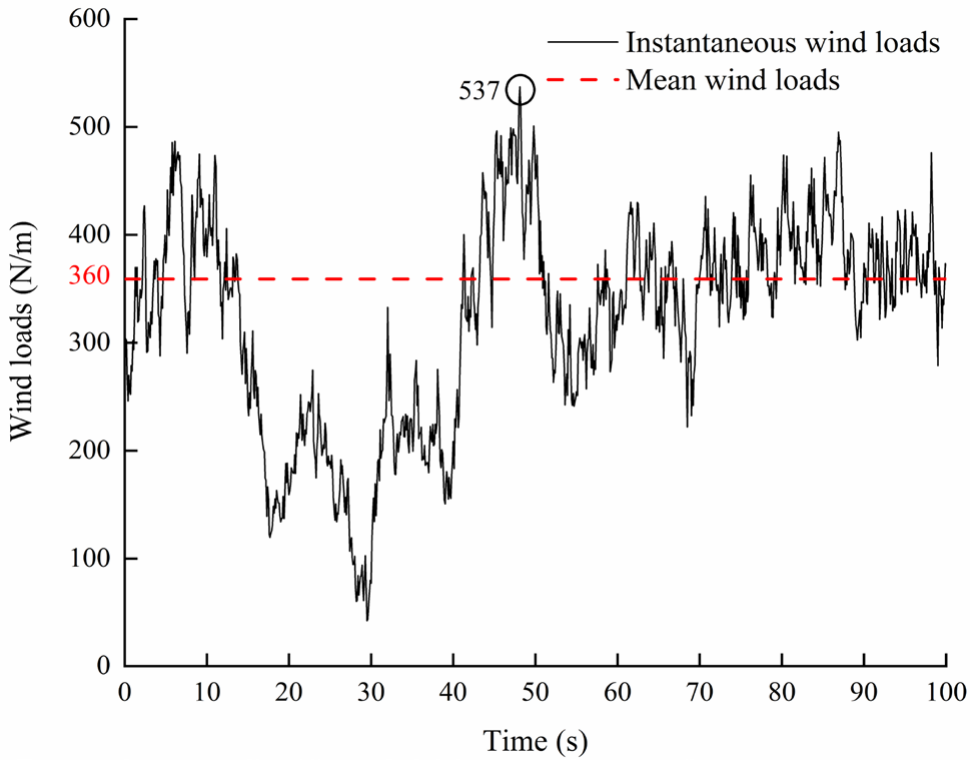
Wind loads acting on greenhouse.

The average wind pressure *W*_1_ under the action of average wind is 360 N/m^2^. The instantaneous wind pressure *W*_2_ under the action of instantaneous wind is calculated as follows^25^:

4 $$ W_{2} = 0.5\rho V(x,y,z,t)^{2} $$

where *W*_*2*_ is instantaneous wind pressure, N/m^2^; *ρ* is air density, kg/m^3^; *V *(*x, y, z, t*) is instantaneous wind speed, m/s. The wind pressure of the two load cases is shown in Fig. 5. From Fig. 5, we can see that the maximum instantaneous wind load can reach 537 N/m, which is higher than the mean wind load. Therefore, it may underestimate the actual responses of greenhouse structure under strong wind.

**Figure 6:**
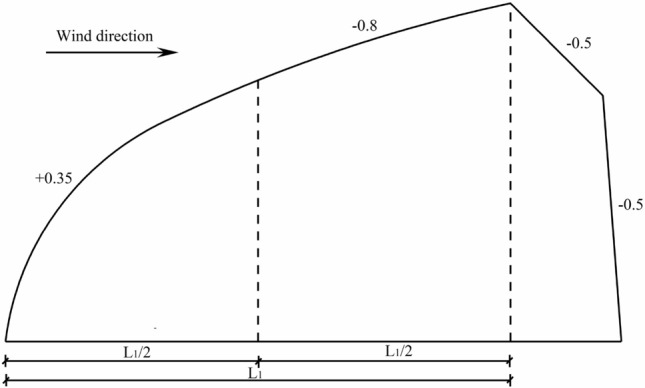
Shape coefficient of wind load on the greenhouse.

When calculating the main structure of greenhouse, the standard value of wind load is calculated as follows^25^:

5 $$ W_{{\text{k}}} = \mu_{{\text{z}}} \mu_{{\text{s}}} W $$

where *W*_*k*_ is standard value of wind load, N/m^2^; *μ*_*z*_ is wind pressure variation coefficient for adjusting the wind speed along the height and is taken to be 1.0 in the calculations; *μ*_*s*_ is shape factor of wind load, which is related to the ratio of rise to span, the values of *μ*_*s*_ at different parts are given in Fig. 6 for the transverse direction of wind load; *W* is the wind pressure, N/m^2^. From Fig. 6, we can see that most parts of the greenhouse structure are subjected to wind suction, where ‘- ‘ sign stands for wind suction and ‘ + ’ sing stands for wind pressure.

## Results and discussion

### Validation of the finite element model

In order to ensure the effectiveness of the ANSYS finite element analysis method used in this study, the analysis results are compared with the literature. Based on the greenhouse load standards from five countries, Kim et al.^26^ analyzed that the bending moment distribution of single span greenhouse under the combined action of wind load and self-weight by using SAP2000. With the same boundary and load conditions, the greenhouse structure is analyzed by using the modeling method described in this study. Table 4 shows the comparison of the maximum bending moment between the present results and those in the literature. The relative deviation of the maximum value of bending moment is less than 2%, which verifies the accuracy and effectiveness of the modeling method in this study.

**Table 4:** Comparison of the maximum bending moment between the present results and those in the literature.

Load case	1	2	3	4	5
Literature Present	0.28 0.28	0.64 0.65	0.13 0.13	0.94 0.96	0.77 0.78

### Vibration characteristics of greenhouse structure

**Figure 7:**
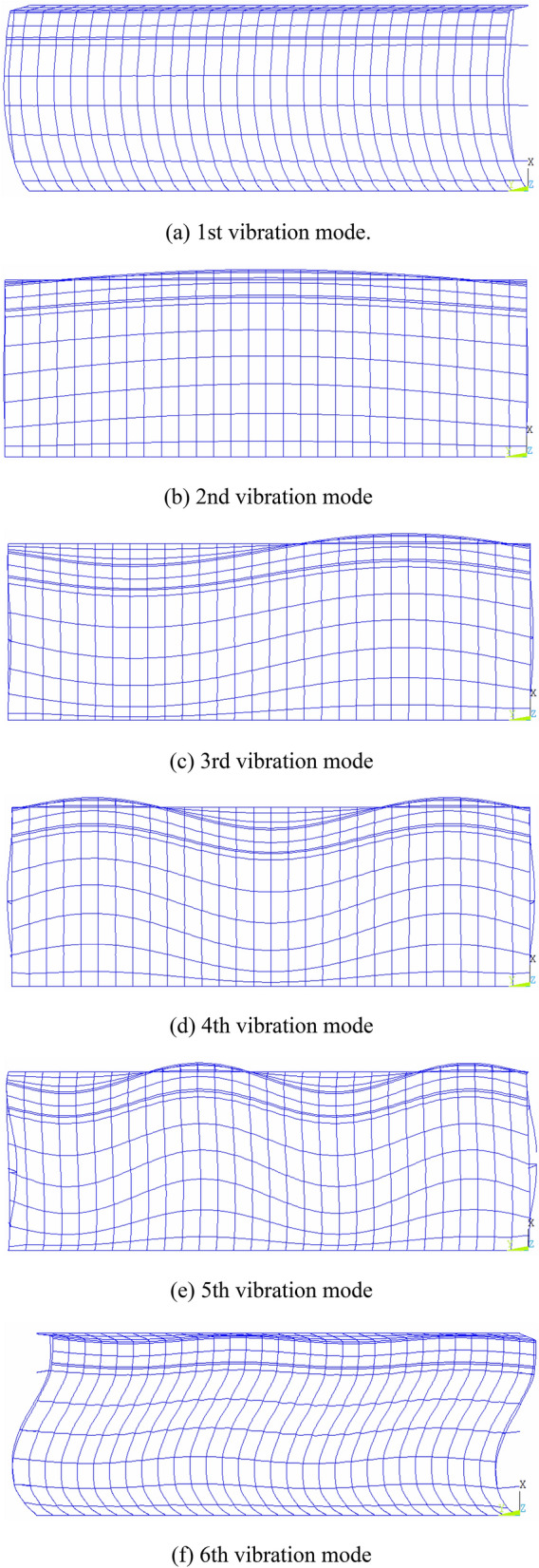
Six mode shapes of the greenhouse.

Modal analysis is used to determine the vibration characteristics of the structure, including two important parameters of the structure: the natural frequency (period) and the array of each order, which also can be collectively referred to as the modal of the structure. According to the vibration theory, for a multi degree of freedom vibration system, the low order natural frequency of the system has a great influence on the dynamic response of the system. With the increase of structural frequency, the contribution of each mode in dynamic characteristics will decrease rapidly. Thus, for multi degree of freedom systems, only the low order natural frequency can reflect the dynamic characteristics of the system. The first six modal frequencies and modes of greenhouse structure are obtained by Lanczos vector iteration method. Table 5 lists the first six natural frequencies of the greenhouse structure, and Fig. 7 shows the vibration modes of the first six modes. The first vibration mode is *y*-axis translational vibration mode; the second vibration mode is *x*–*z* axis bending vibration mode; the third vibration mode to the sixth vibration mode is torsional vibration mode.

**Table 5:** First six natural frequencies of the structure.

Mode order	1	2	3	4	5	6
Frequency/Hz	2.9585	3.3653	3.7718	4.3959	5.1455	5.5600

### Stress analysis

#### Axial stress

**Figure 8:**
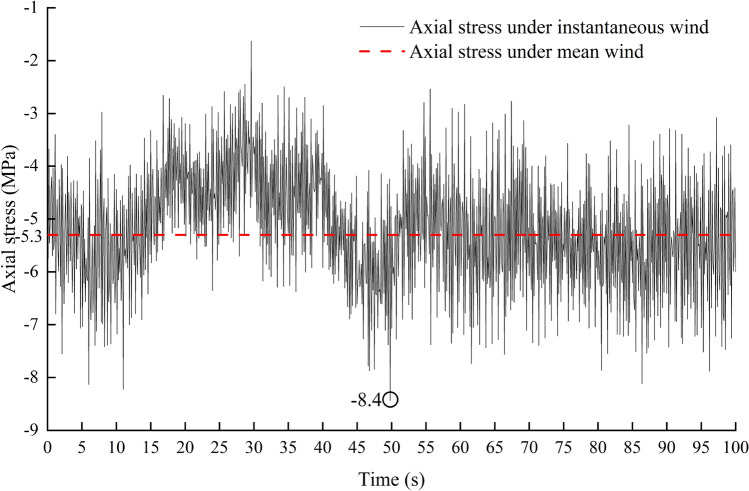
Stress time history of the element with maximum axial stress.

Through the time history analysis of load case (2), the results are shown in Fig. 8. The time history change is consistent with that of wind pressure. The maximum axial stress is 8.4 MPa at 49.8 sand the maximum axial force element is located on the end column. The static analysis of load case (1) shows that the maximum axial stress is 5.3 MPa, and the position of the maximum is consistent with load case (2). All the elements are subject to axial pressure stress. The axial stress of greenhouse structure is much lower than the yield strength of material under two load cases.

#### Bending stress

**Figure 9:**
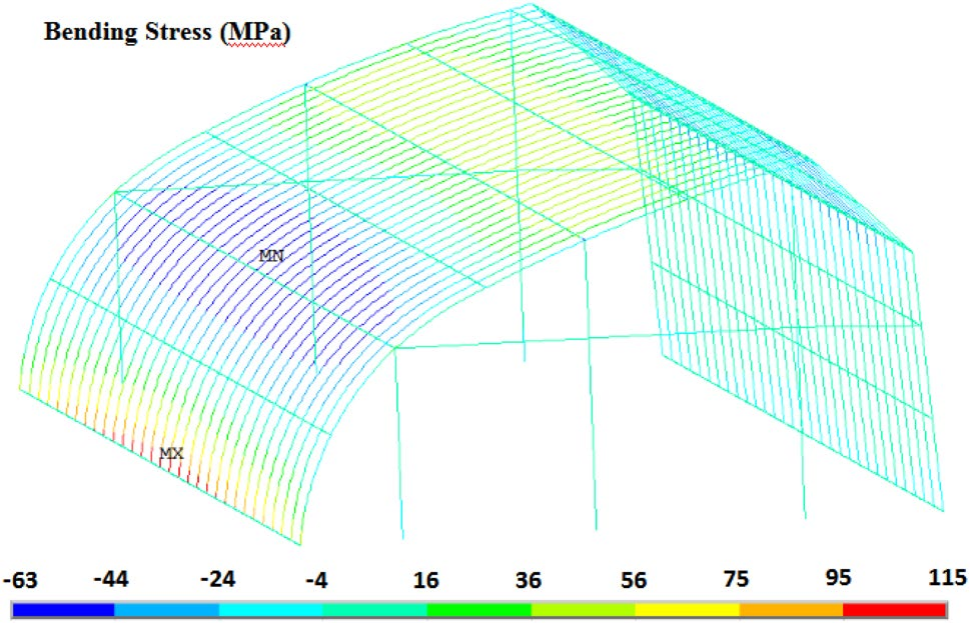
Bending stress of greenhouse structure under instantaneous wind.

**Figure 10:**
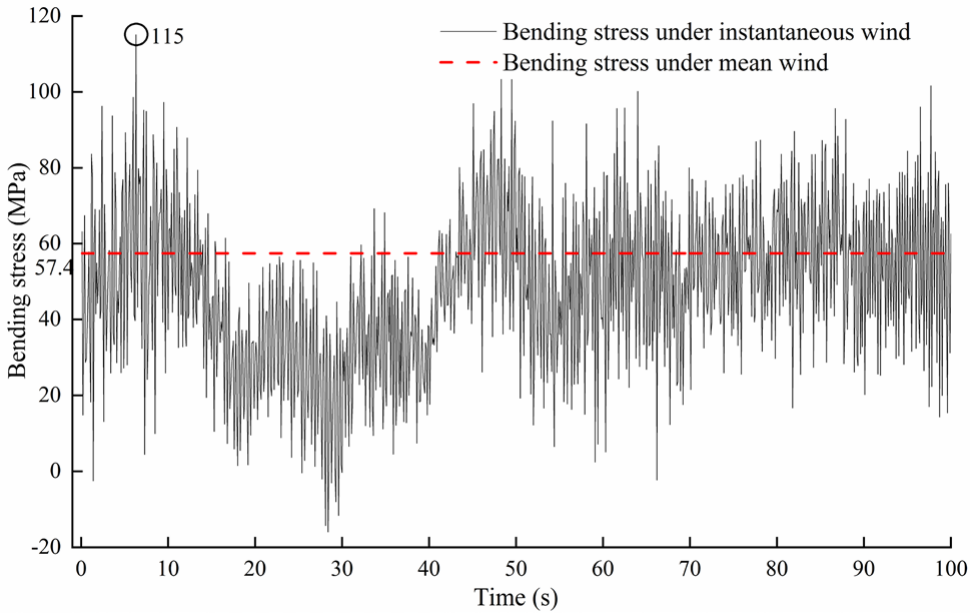
Stress time history of the element with maximum bending stress.

**Figure 11:**
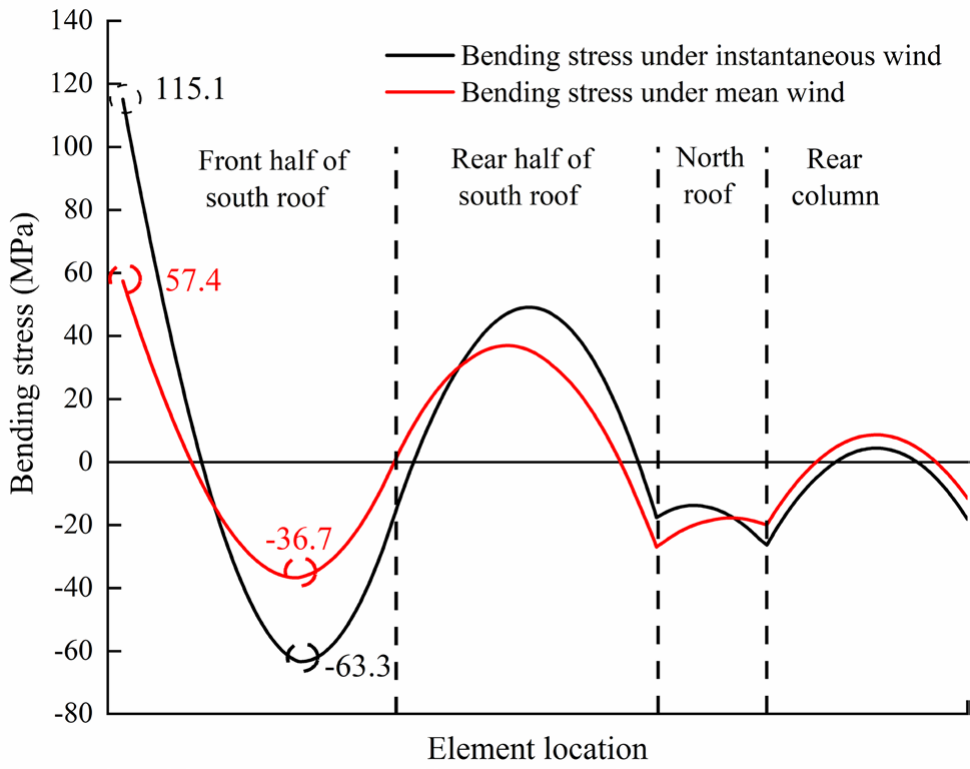
Bending stress distribution of the 16th arch from East.

Figure 9 shows the overall bending stress distribution of the greenhouse frame under load case (2). The maximum bending stress element is located in the bottom corner of the south side of the 16th arch (the middle position of the greenhouse) on the east side of the greenhouse. Because the end of the frame is welded on the embedded part of the foundation, these local rigid discontinuities will produce stress concentration^7^. Therefore, more attention should be paid to the phenomenon of stress concentration at the foundation. Due to the existence of end columns, the bending stress distribution in the middle frame is different from than end frame. Therefore, it is necessary to conduct 3-D full-scale analysis. The time history curve of bending stress of the element is shown in Fig. 10. From Fig. 10, we can see that the time history of bending stress change trend is consistent with that of wind pressure. The maximum bending tensile stress is 115 MPa occurred at 6.3 s, which is higher than that under average wind load with a value of 57.4 MPa. The stress distribution of the 16th arch on the east side under two wind load cases is analyzed. The results are shown in Fig. 11. The bending stress distribution characteristics of the two wind load cases are basically similar. The maximum bending stress appears at the south bottom corner of the arch frame. The maximum bending stress under mean wind load is 57.4 MPa. Except for the south end of the arch, the bending stress of one third and two thirds of the south roof of the greenhouse is larger, and the stress of the north roof and the rear column of the greenhouse is smaller. At the joint between south roof and north roof, north roof and rear column, the bending stress is greater than that at both sides. The maximum bending stress under instantaneous wind load is about 2 times higher than that of under mean wind load.

### Deformation analysis

**Figure 12:**
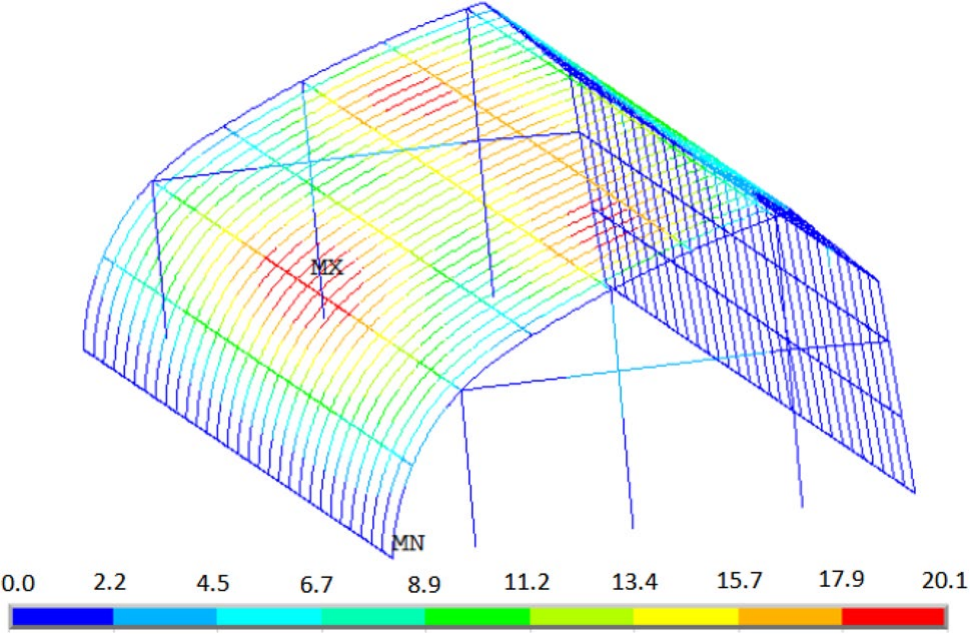
Displacement of greenhouse structure under instantaneous wind.

**Figure 13:**
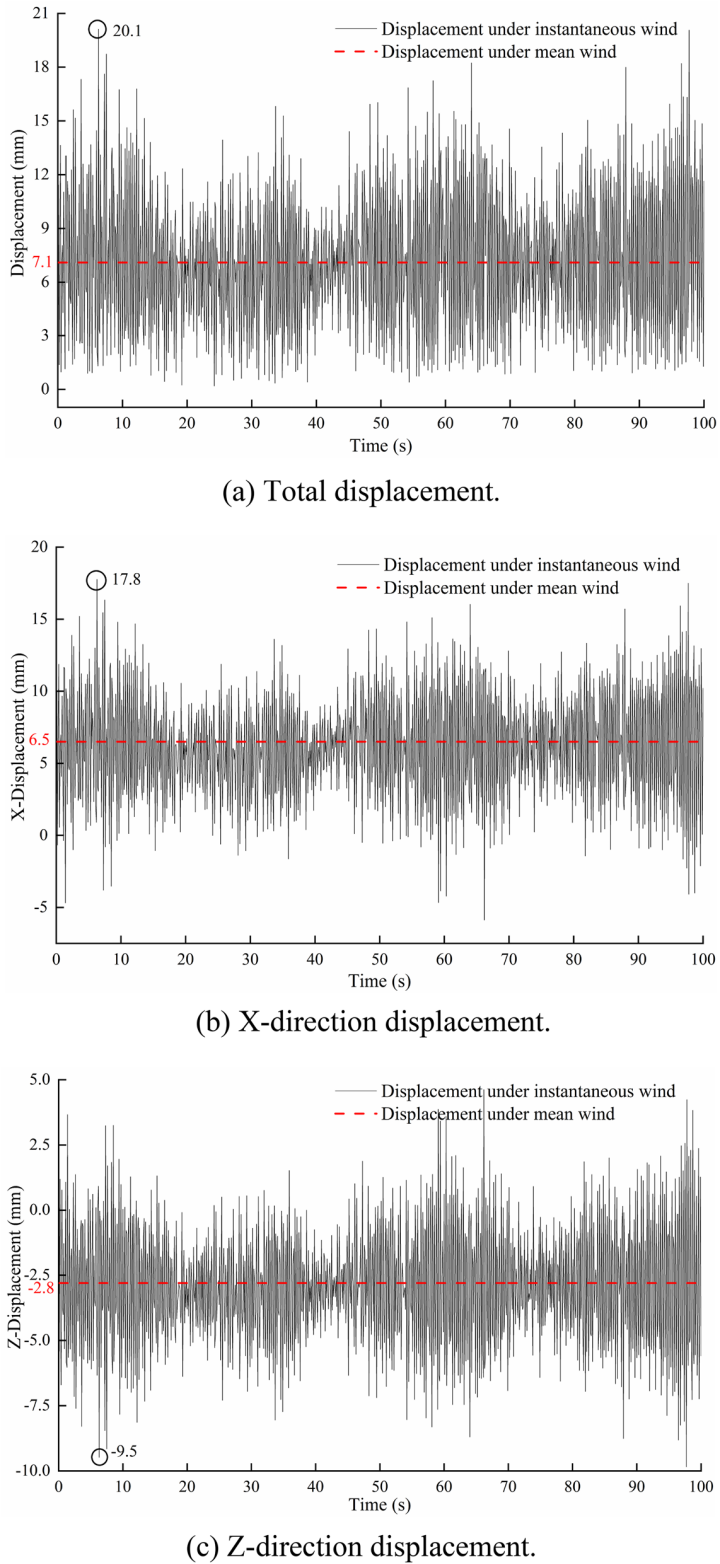
Displacement time history of the maximum displacement node.

**Figure 14:**
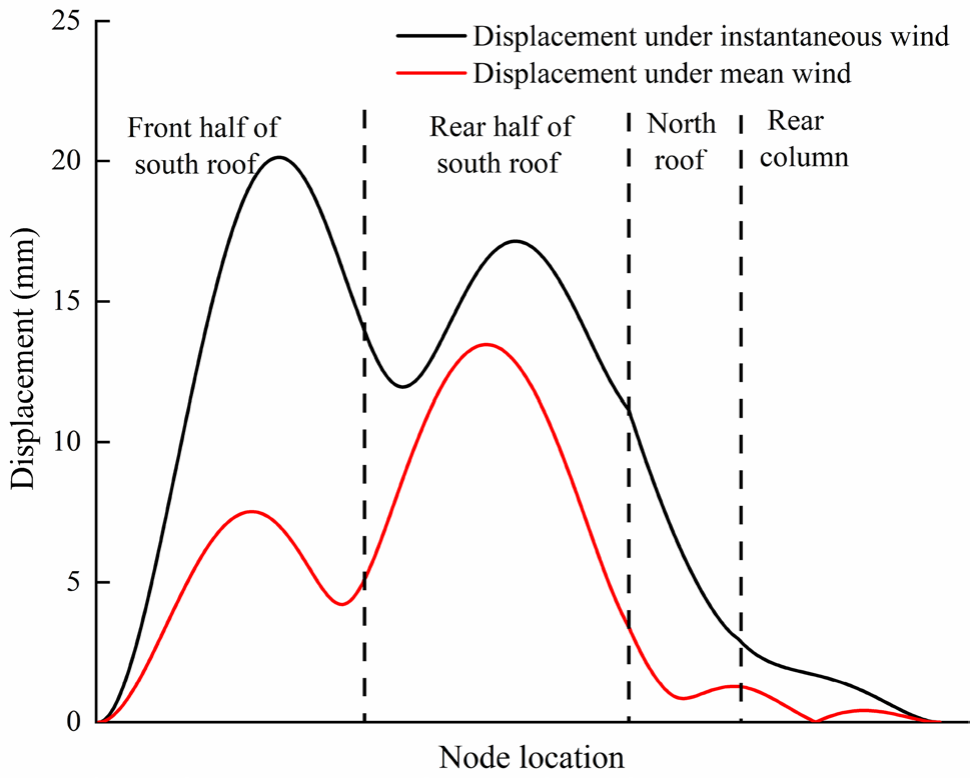
Displacement distribution of the 11th arch on the east side.

Figure 12 shows the displacement distribution of the greenhouse structure under load case (2). The overall displacement of the south roof of the greenhouse is large. The maximum displacement node is located at the height of 2.9 m of the 16th arch (the middle position of the greenhouse) on the east side of the greenhouse. The displacement time history curve of the node is shown in Fig. 13. The maximum displacement occurs at 6.3 s, which is the same as the maximum bending stress. The maximum displacement is 20.1 mm, the horizontal displacement component is 17.8 mm, and the vertical displacement component is -9.5 mm. Correspondingly, the displacement of the joint under load case (1) is 7.1 mm, the horizontal displacement component is 6.5 mm, and the vertical displacement component is -2.8 mm. The displacement distribution of the 16th arch on the east side under the two load cases is analyzed. The results are shown in Fig. 14. The displacement distribution characteristics of the two load cases are basically the same. There are two peaks in the displacement curves of the two load cases, and they are located on the south roof of the greenhouse. In case (1), the two peak values of displacement appear at the height of 2.6 m and 4.7 m respectively, and the maximum displacement is 13.4 mm. In case (2), the two peaks of displacement appear at the height of 2.9 m and 4.9 m respectively, and the maximum displacement is 20.1 mm.

### Displacement wind vibration coefficient of greenhouse

The dynamic response analysis of structures caused by fluctuating wind is more complex than static analysis. In structural design, dynamic load is converted into equivalent static load by wind vibration coefficient. Displacement wind-induced vibration coefficient is an effective wind-induced vibration coefficient, which can be expressed by the ratio of maximum displacement response to average displacement response. The displacement wind-induced vibration coefficient can be calculated as follows^27^:

6 $$ \beta = \frac{{U_{t} }}{{U_{m} }} = 1 + \frac{{U_{i} }}{{U_{m} }} = 1 + \frac{{\mu \sigma_{i} }}{{U_{m} }} $$

where *U*_*t*_ is the total displacement; *U*_*m*_ is the average displacement caused by average wind loads; *U*_*i*_ is the fluctuating displacement caused by fluctuating wind loads; *μ is* peak factor or guarantee factor, and the value is 3.5 according to the specification; *σ*_*i*_ is mean square error of the response and can be calculated as follows^27^:

7 $$ \sigma_{i} = \sqrt {\frac{{\sum\nolimits_{i = 1}^{n} {(U_{ti} - U_{m} )^{2} } }}{n - 1}} $$

In this structure, the wind-induced vibration coefficient of the displacement node is 2.02. Therefore, it will underestimate the responses of greenhouse structure under strong wind. It is suggested that when design greenhouse, it better to double the average wind load to account for fluctuating wind load.

## Conclusions

In this study, a 10 m span whole steel frame solar greenhouse was taking as the research objective. The harmonic superposition method was used to simulate the wind speed time history. The response characteristics of greenhouse under average wind load and instantaneous wind load were analyzed. The results are as follows:

(1) Under wind loads, the whole steel frame solar greenhouse mainly bears bending stress, and the axial pressure stress is relatively small. The maximum bending stress occurs at the end of south roof. The maximum bending stress is lower than the yield strength of the material. The maximum displacement occurs on the south roof at the height of 2.6–2.9 m.

(2) The bending stress, axial stress and displacement of greenhouse skeleton under average wind loads are lower than those under instantaneous wind loads. Therefore, it is necessary to consider the influence of fluctuating wind load in the structural safety design of greenhouse.

(3) To improve the efficiency of calculation and design, a wind-induced vibration coefficient of 2.02 was proposed to considering the effects of fluctuating wind load on the structural dynamic responses.

This study investigated the dynamic responses of whole steel frame solar greenhouse under wind loads. However, the effects of plastic film on the structure safety of greenhouse under wind loads does not consider. In reality, the plastic film may be failed before the skeleton structure. Therefore, the fluid–solid coupling method should be used to consider plastic film on the dynamic responses of greenhouse under wind loads in the future work.
